# Effectiveness of a Mindful Compassion Care Program in reducing burnout and psychological distress amongst frontline hospital nurses during the COVID-19 pandemic: a study protocol for a randomized controlled trial

**DOI:** 10.1186/s13063-022-06666-2

**Published:** 2022-09-02

**Authors:** Luca Bodini, Chiara Bonetto, Simone Cheli, Lidia Del Piccolo, Michela Rimondini, Alberto Rossi, Angela Carta, Stefano Porru, Francesco Amaddeo, Antonio Lasalvia

**Affiliations:** 1 Section of Psychiatry, Department of Neuroscience, Biomedicine and Movement Sciences, University of Verona, Policlinico “G.B. Rossi, ” P.le Scuro, 10, 37134 Verona, Italy; 2 Center for Psychology and Health, Tages Charity, Florence, Italy; 3 School of Human Health Sciences, University of Florence, Florence, Italy; 4 Section of Clinical Psychology, Department of Neuroscience, Biomedicine and Movement Sciences, University of Verona, Italy and USD Psicologia Clinica BR, Azienda Ospedaliera Universitaria Integrata (AOUI) di Verona, Verona, Italy; 5 Department of Mental Health, ULSS9 Scaligera, Verona, Italy; 6 Section of Occupational Medicine, Department of Diagnostics and Public Health, University of Verona and UOC Medicina del Lavoro, Azienda Ospedaliera Universitaria Integrata (AOUI) di Verona, Verona, Italy; 7 UOC Psicosomatica e Psicologia Medica, Azienda Ospedaliera Universitaria Integrata (AOUI) di Verona, Verona, Italy; 8 UOC Psichiatria, Azienda Ospedaliera Universitaria Integrata (AOUI) di Verona, Verona, Italy

**Keywords:** COVID-19, Mindfulness, Compassion, Burnout, Healthcare workers, Nurse

## Abstract

**Background:**

Recent studies have shown that nurses have been more affected by the COVID-19 pandemic than any other group of hospital workers in terms of anxiety, depression, and burnout. Several clinical studies had previously demonstrated the effectiveness of mindfulness and compassion interventions in reducing burnout and emotional distress amongst healthcare professionals.

**Methods and analysis:**

A parallel-group randomized controlled trial will assess the feasibility, acceptability, and efficacy of a mindfulness and compassion-focused programme on frontline nurses who had been working during the COVID-19 pandemic. Seventy-two participants will be recruited from Verona University Hospital Trust (Veneto Region, north-east Italy) and will be divided equally into an intervention group and a control group. Primary outcome will be assessed using the Emotional Exhaustion subscale of the Maslach Burnout Inventory General Survey (MBI-GS). Secondary outcomes will be measured by the Cynicism and Professional Efficacy subscales of the MBI-GS, the Patient Health Questionnaire (PHQ-9), the Generalized Anxiety Disorder (GAD-7), the Insomnia Severity Index (ISI), the Impact of Stressful Events (IES-R), the Perceived Stress Scale (PSS), the Five Facet Mindfulness Questionnaire (FFMQ), and the Forms of Self-Criticising/attacking and Self-Reassuring Scale (FSCRS).

**Discussion:**

The study aims to fill a gap in the literature and present a scientifically validated intervention for those healthcare professionals most exposed to the stressful conditions of working during the COVID-19 pandemic.

**Trial registration:**

ClinicalTrials.gov; Identifier: NCT05308537

## Introduction

### Background and rationale

From the beginning of the COVID-19 pandemic, healthcare workers (HCWs) worldwide experienced overwork, increased health risks in the absence of clear guidelines, and the reorganization of their activities. These factors, combined with exposure to a condition of increased mortality and a sense of uncontrollability, have led to growing incidences of burnout, anxiety, depressive symptoms, and a reduced investment of energy in the professional sphere [1–3]. Nurses were amongst the HCWs most exposed to the negative psychological effects of the COVID-19 pandemic, particularly those working in departments most involved in the treatment of COVID-19 patients, such as intensive care, infectious disease, and pulmonary units and emergency rooms [1, 2, 4].

Currently several groups across the world are working to develop initiatives aimed at supporting the well-being of HCWs facing the psychological impacts of the COVID-19 pandemic, through easy-to-access supportive psychological services [5–7]. However, there is a lack of evidence from randomised trials testing interventions with a sound base of evidence that can inform the selection of treatments that may be beneficial to the mental health of frontline HCWs facing the current pandemic [8].

Previous research has shown the effects of certain intervention strategies in reducing burnout and emotional distress amongst HCWs in the pre-COVID 19 era. These interventions included mindfulness, stress management, and small group discussion [9–13]. Over the past 20 years, studies have shown that mindfulness-based interventions are effective in reducing stress, anxiety, depression, and obsessive disorders [14–18]. Mindfulness can be defined as an awareness of present-moment thoughts, feelings, and bodily sensations through intentional and non-judgemental attention [19]. It is a state of consciousness that allows the mind and body to build a relationship of harmony and balance, a predisposing element for the condition of well-being [14]. The most scientific mindfulness-based interventions, such as MBSR (mindfulness-based stress reduction) [19] and MBCT (mindfulness-based cognitive therapy) [20] consist of weekly group meetings of 2 h and 30 min for a period of 8 weeks, with an intensive day in the middle of the programme.

Studies investigating the diffusion of clinical models based on mindfulness have allowed us to hypothesize that it can play an important role in emotional self-regulation [21]. It can, therefore, be argued that mindfulness allows one to reduce suffering and create a healthy mind by using self-processing processes [22] and modulating awareness of self and one’s emotional states and behaviours, as well as relationships with others. The ability to manage suffering, for example of patients and caregivers, stems from an empathic and emotional attunement known as compassion to the suffering of another person [23]. Compassion can be defined as the awareness of one’s own and others’ suffering and the intention or motivation to alleviate it [24]. It has been associated with the capacity to adjust to distressful experiences during dramatic events such as pandemics [25, 26] and amongst HCWs in particular [27, 28]. From this compassion-focused point of view, the distress a HCW may experience may be targeted and reduced by balancing the capacity to be compassionate with the others together with the capacity to receive compassion from oneself and others [25, 29]. This theoretical stance is consistent with the clinical practice of supporting HCWs in finding a sustainable work-life balance, promoting teamwork and valuing their professional commitment in caring their patients.

Compassion arises when an individual feels a deep connection to the suffering of others [30]. It facilitates emotional regulation, psychological flexibility and promotes relational and ethical skills [22, 30, 31]. Several studies have indicated that efficacious results can be achieved through 4-week or 6-week programmes [32–34]. These seem to be equally effective as longer programmes in terms of reducing burnout and improving the well-being and quality of life of HCWs. They are also more easily implemented in organizational settings, are less costly, and have a higher likelihood of participation and a lower risk of dropout [18].

Studies evaluating the efficacy of mindfulness amongst HCWs working during the current COVID-19 pandemic are rare, though some are in progress [11]. Results from mindfulness protocols lead us to hypothesize that such interventions may be a valuable therapeutic tool for HCWs who are working under pandemic conditions and are at risk of experiencing burnout and psychological problems. The present project is based on the results of a longitudinal study that assessed the psychological impact of the COVID-19 pandemic on HCWs at the Verona University Hospital Trust during the lockdown phase (March-May 2020) and after 1 year later [2, 4]. The study found a good number of hospital staff displayed clinically significant symptoms of post-traumatic distress, anxiety, depression, and burnout; moreover, hospital staff, particularly nurses and those working in intensive care units and COVID-19 sub-intensive wards, experienced a further increase in burnout and depression after 1 year [4]. The findings indicate the need to implement and test an intervention study aimed at mitigating the sustained psychological impact of the pandemic on those at greater risk of adverse psychological outcomes.

The present project proposed here aims to implement and evaluate the feasibility, acceptability, and effectiveness of a preliminarily tested intervention [32, 35] that integrates the most widely used and scientifically validated mindfulness protocols such as mindfulness-based stress reduction [19], mindfulness-based cognitive therapy [17, 20], and compassion-focused therapy [30], in a healthcare context (the Verona University Hospital Trust) during the COVID-19 pandemic. The purpose of the intervention is to reduce burnout and psychological distress amongst frontline nurses involved in the clinical management of COVID-19 patients.

## Methods

### Trial design

A randomized controlled trial (RCT) parallel group waiting list design will be used to assess the efficacy of a mindful and compassion programme for nurses (superiority trial) (Table 3 in Appendix). Participants will be randomly assigned to one of two groups: the experimental group will receive the Mindful Compassion Care Program (MCCP) [32, 35], and the control group will be allocated to a waiting list (WL) with an allocation ratio of 1:1. The effectiveness of the experimental intervention will be assessed by comparing the changes in the level of burnout at the end of the treatment and after 1-month later (this follow-up interval has been set due to organizational and time constraints). Other psychological dimensions will also be investigated (i.e. anxiety and depressive symptoms, posttraumatic symptoms, insomnia, perception of stress, mindfulness skills and evaluation of self-criticism and self-reassurance).

### Study setting

The study will be conducted at the Verona University Hospital Trust (Azienda Ospedaliera Universitaria Integrata [AOUI]), the second-largest hospital in Italy in terms of the number of beds and the fifth largest in terms of admissions. The trust employs 6000 people, including nearly 2000 nurses. On 17 March 2020, the Veneto regional government converted part of the hospital into a COVID-19 hospital. Dedicated pathways for both suspected and confirmed COVID-19 cases were established within the hospital, as well as in other hospital units located in clearly restricted areas devoted to the treatment of COVID-19 patients.

### Eligibility criteria

The population under investigation comprises frontline nurses who have been engaged over the two years of the pandemic in the clinical management of the most severe or critical COVID-19 cases (chief nurses and nurse managers will not be involved). Nurses will be recruited from intensive care units (ICUs) and sub-intensive COVID-19 wards (i.e. infectious disease unit, pulmonary medicine, internal medicine units converted to COVID-19 units). Eligible participants will be contacted by email by the research group and will receive an invitation to participate voluntarily.

To be included in the study, a participant/s will have to be (1) a nurse employed at AOUI for the past 2 years; (2) working within ICUs, infectious disease unit, and pulmonary medicine and internal medicine units that have been converted to COVID-19 sub-intensive units; and (3) scoring above the cut-off score for the Emotional Exhaustion sub-scale (EX) of the MBI-GS (equal to or greater than 2.20) in accordance with Italian norms [3].

Respondents will be excluded if they (1) have participated in mindfulness-based interventions in the previous 6 months; (2) show a score < 2.20 in the EX subscale of the MBI-GS; or (3) are receiving psychosocial or psychiatric treatment. Participants allocated to the WL will be requested not to participate in a mindfulness course offered elsewhere.

## Interventions

The MCCP comprises six regular 1 h and 30-min sessions and 1 all-day class lasting 4 h and 30 min. The intervention is a proven effective mindfulness programme [32, 35] based on well-known scientific programmes such as mindfulness-based stress reduction (MBSR) [19], mindfulness-based cognitive therapy (MBCT) [20] and compassion-focused therapy (CFT) [30]. It was pilot-tested in a non-randomized controlled trial of nursing students (*n* = 82) and exhibited medium to very large effect sizes (with Cohen’s *d* ranging from 0.57 to 1.25) in changes in burnout symptoms between both pre- and post-assessment in the experimental group, as well as control and experimental group in the post-assessment [32]. Two recent cases studies of nurses recruited during the pandemic showed reliable changes in several outcomes by the end of the intervention [35].

The MCCP consists of a theoretical component and standard mindfulness practices (i.e. mindfulness meditation exercises) along with a few bespoke practices. It aims to explain the foundations and the applications of a more mindful and compassionate approach to healthcare. It has three specific objectives: (a) to explore the concept of reflexivity as a tool in monitoring and revising nursing practice and illness experience, (b) to explain useful strategies that prevent burnout and promote engagement, and (c) to explain useful strategies that facilitate and foster compliance in patients [36]. The purpose of the intervention is to reformulate these general objectives through the lens and within the framework of mindful compassion.

The practice of mindfulness involves a series of formal exercises led by an experienced instructor. The participant will learn to bring their attention to the present moment through an awareness of their body, breathing, and senses; gradually relate to their inner and external experience in a more welcoming and non-judgmental way; and carry out informal exercises (e.g. they learn to extend to different moments in their day a the state of present-orientated awareness that they experience during their formal practices, e.g. by paying full attention to what they are doing at a certain point in time). Daily home practice is conducted through guided audio tracks and the completion and reading of a number of worksheets and materials.

Course activities will include mindfulness exercises led by an instructor that can be carried out every day, yoga practices with gentle body movements that are accessible to all, and group dialogue. Table 1 shows how the programme is to be structured.

**Table 1:** Themes and programme of the MCCP

	Mindfulness exercises	Mindfulness psychoeducation
Week 1	Automatic pilot; soothing breath; standing yoga	Mindfulness practice and programmes; automatic pilot
Week 2	Automatic pilot: the day I suffered; soothing breath	Mind-wandering; thoughts and feelings
Week 3	Automatic pilot; experiential acceptance; standing yoga	Automatic thoughts; acceptance
Week 4	Soothing breath; compassionate self	Psychological flexibility; compassionate mind
Week 5	Sitting meditation; three pillars of death; mindful walking	Self-compassion; loving kindness
Week 6	Sitting meditation; loving kindness; compassion flowing out	Compassionate care
Final Session	Automatic pilot; sitting meditation; sitting yoga; breath meditation; mindful walking; loving kindness; compassion flowing in; compassion flowing out; breathing group	Summary

Exercises conducted during the intervention programme include a body exploration exercise, which consists of focusing the attention on different parts of the body (e.g. toes, back, and head) and physical sensations (e.g. pain or muscle tension) in the present moment; breath awareness meditation, which consists of turning the attention to the breath, noticing how bodily sensations change during inhalation and exhalation; and slow walking meditation, in which participants become aware of their steps, from beginning to end. Throughout, participants will be trained to maintain an attitude of acceptance, non-judgementalism, and equanimity, and foster attention, awareness, recognition, and emotional regulation. The mindful compassion sessions will allow participants to share their experience of practice or difficulties they have encountered. These will be followed by feedback from the teacher. The course will have a maximum of 12 participants.

If participants of either group report any adverse events during the trial, this will be registered in the electronic database system. Serious adverse events will be reported to the ethics committee. If participants require additional mental health support, they will be referred to the regular services, but it is also possible for participants to consult with an appointed psychologist if they so request or if the trainer so advises.

Each session will focus on topics that will be explored through specific exercises, presented by a licensed psychologists with demonstrated experience in the application of mindfulness-based protocols and who meets the criteria of the internationally agreed good practice guidelines of the UK network for mindfulness-based teachers.

**Fig. 1:**
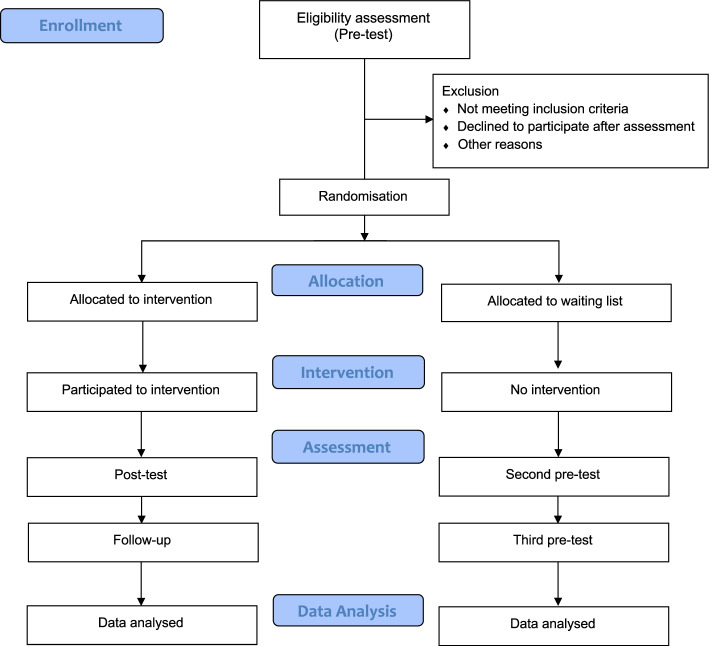
Flowchart of the trial design

If a given participant at the end of the trial continues to display high or higher levels of distress, they will be referred to the Psychiatric Consultation Service at the UOC of Psychosomatics, Verona Hospital Trust. This service has provided an individual help pathway for HCWs with psychological distress since the beginning of the pandemic. The WL is the control group planned for this study. It will remain in place for the entire period of the intervention. Details on the rationale of the study are shown in the flowchart (Fig. 1).

### Outcomes

As the proposed intervention aims to mitigate the prolonged strain on hospital staff by working with the most severe or critical COVID-19 patients, burnout—a specific indicator of chronic work-related dysfunctional response—was selected as the main outcome of interest. In particular, any change in the sub-subscale of emotional exhaustion (EX) between pre-treatment and 1-month post-treatment was chosen as the primary outcome of burnout [37]. Emotional exhaustion is a psychological construct that explores the perception of physical and emotional fatigue. It is characterized specifically by a lack of energy required to handle daily life and the prevalence of feelings of apathy and emotional detachment at work. It is the central dimension in burnout [38] and correlates strongly with job performance, absenteeism, higher psychopathological risk, and poor organizational effectiveness [39]. Numerous studies have found EX in HCWs to be associated with acute depressive symptoms, anxiety disorders, sleep problems, and poor quality relationships with patients [1, 33–35].

The secondary outcomes of the intervention will be to observe any change between pre-treatment and post-treatment and between pre-treatment and 1-month post-treatment, in:

- Professional efficacy (MBI-GS) [37]
- Cynicism (MBI-GS) [37]
- Depressive symptoms (PHQ-9) [40]
- Anxiety symptoms (GAD-7) [41]
- Post-traumatic symptoms (IES-R) [42]
- Insomnia (ISI) [43]
- Perception of stress (PSS) [44]
- Mindfulness skills (FFMQ) [45]
- Evaluation of self-criticism and self-reassurance (FSCRS) [46]

The assessment instruments to be used are as follows:

- Personal socio-demographic information will be collected by using an ad hoc schedule, addressing personal (gender, age, having had psychological problems developed before the COVID-19 outbreak, having received psychological treatment other than mindfulness over the past 2 years) and job-related characteristics (place of work within hospital, working experience, monthly night shifts).
- The Maslach Burnout Inventory–General Survey (MBI-GS) is a self-rated scale that explores the individual’s relationship with work with respect to burnout in particular. It is a modified and shortened version of the original MBI [37] and consists of 16 items and three subscales: emotional exhaustion (5 items), which measures feelings of being overextended and exhausted by one’s work; cynicism (5 items), which measures an indifference or a distant attitude towards your work; professional efficacy (6 items), which measures satisfaction with past and present accomplishments, and it explicitly assesses an individual’s expectations of continued effectiveness at work. Responses to the MBI-GS items are on a 6-point Likert scale, ranging from 0 (*never*) to 6 (*always*).
- Patient Health Questionnaire (PHQ-9) is a nine-question self-report measure of depression symptoms. It asks about the American Psychiatric Association’s Diagnostic and Statistical Manual (DSM) nine diagnostic symptoms of major depressive disorder and scores on each symptom range from 0 (*not at all*) to 3 (*nearly every day*) [40].
- Generalized Anxiety Disorder (GAD-7) is a self-rated questionnaire consisting of seven items that investigate the level of anxiety and worry felt by the respondent over the previous 2 weeks [41].
- Insomnia Severity Index (ISI) is a seven-item questionnaire that asks respondents to rate the nature and symptoms of their sleep problems [43].
- Impact of Events Scale – Revised version (IES-R) is a 22-item self-report that assesses subjective distress caused by traumatic events [42]. The IES-R contains seven additional items related to the hyperarousal symptoms of PTSD that were not included in the original IES. Respondents are asked to identify a specific stressful life event and indicate how distressed or bothered they had been during the previous 7 days according to each “difficulty” listed. Items are rated on a 5-point scale ranging from 0 (*not at all*) to 4 (*extremely*). The IES-R yields a total score (ranging from 0 to 88). Subscale scores can be calculated for the intrusion, avoidance, and hyperarousal subscales.
- Perceived Stress Scale (PSS) is a 10-item questionnaire to measure the degree to which life situations are appraised as stressful. Psychological stress has been defined as the extent to which persons perceive (or appraise) that demands upon them exceed their ability to cope [44].
- The Five Facet Mindfulness Questionnaire (FFMQ) is a self-report measure consisting of 39 items based on a five-facet model (i.e. observe, describe, act with awareness, nonjudgement, and nonreaction) [45]. It is widely used for dispositional mindfulness [14].
- Forms of Self-Criticising/attacking and Self-Reassuring Scale (FSCRS) is a self-administered tool for the assessment of three forms of self-to-self relating as a process measure [46]. Two subscales represent maladaptive forms of self-to-self relating, namely, self-criticism induced by the desire to correct or improve certain aspects of the self—which is referred to as inadequate self (IS)—and self-criticism arising from the desire to hurt, persecute, and attack the self—which is referred to as hated self (HS). A third subscale, RS, reflects the ability to reassure oneself. The questionnaire consists of 22 items.

### Participant timeline

Information on enrolment, intervention, and assessment in the trial can be found in Table 2.

**Table 2:** Enrolment, interventions, and assessments of the MCCP

	Study period				
	Enrolment	Allocation	Pre-treatment	Post-treatment	Follow-up
	− *T*_1_	*T*_0_	*T*_1_	*T*_2_	*T*_3_
Eligibility screening	X				
Informed consent	X				
Randomization		X			
Allocation		X			
Socio-demographics		X			
Job-related characteristics		X			
**Interventions**					
Treatment			X	X	
Waiting list			X	X	
**Assessments**					
Burnout			X	X	X
Depression symptoms			X	X	X
Anxiety symptoms			X	X	X
Post-traumatic symptoms			X	X	X
Insomnia			X	X	X
Perception of stress			X	X	X
Mindfulness skills			X	X	X
Self-criticism and self-reassurance			X	X	X

Primary and secondary outcomes will be measured at baseline (T1), at the end of the intervention (T2), and 1 month after the end of intervention (T3). Adverse events and untoward effects will be assessed during each treatment. Table 1 contains a summary of all the measures in the trial.

### Sample size

Sample size calculation was performed a priori according to the primary outcome on an intention-to-treat basis. A total of 72 nurses (36 nurses per treatment condition) achieves 80% power to reject the null hypothesis of equal means when the population mean difference is − 1.21 with a standard deviation for both groups of 1.8 and a significance level of 0.05 using a two-sided two-sample equal-variance *t*-test (PASS 2021). Both the expected mean change and the standard deviation were found by exploring data (which are available from the authors) regarding the distress of nurses working in COVID-19 wards [1, 4].

### Recruitment

The study description and the invitation to participate will be published in the hospital’s newsletter and will be emailed to the nurses’ address by the Verona Hospital Trust Administration. If the study does not obtain enough responses from the newsletter advert, the research group will organize in-person meetings with all nursing staff working within the selected hospital units to present the study, to explain its rationale and to ask their availability to participate. This may allow to increase the number of potential participants. All nurses who express an interest in participating will receive (via e-mail) an information sheet containing all the project details together with a link to the online screening questionnaire. They will find the participant consent form and the consent form for the use and processing of personal data on the same page. The screening questionnaire will allow us to assess whether the participant meets the inclusion criteria. If they do, they will be added to a temporary list of candidates; if they do not, they will be excluded from the trial. However, those participants not meeting inclusion criteria but requiring psychological support will may seek specialized mental healthcare through the Psychiatric Consultation Liaison Service based at the Psychosomatics Unit within the Verona Academic Hospital Trust.

### Assignment of interventions: allocation

Participants will be randomly allocated to the intervention or the WL control group with an allocation ratio of 1:1. Participants allocated to the experimental group will receive the intervention immediately after randomization, and those assigned to the control group will be offered the same intervention 6 months after. The experimental intervention will take place over 6-weeks. The pre-test, post-test, and 1-month follow-up will be carried out at the same time for both study groups.

The trial statistician will prepare the sequence of treatments randomly permuted into blocks of 2. The randomization schedule will be generated with Stata software (version 17.0; Stata Corp, Corp, College Station, TX, USA) using the ‘ralloc’ command for random allocation of treatments balanced in blocks. The Emotional Exhaustion sub-scale (EX) score of the MBI-GS assessed at the screening phase will be used to perform a stratified randomization based on the median value (EX ≥ median vs. EX< median). Two lists of randomizations will be generated according to the levels of the stratification variable. The trial statistician will communicate by email the randomization results to the responsible for trial, who will notify to the participants by email if they belong to the intervention or the WL control group.

The trial will begin in September 2022. Enrolment will end as soon as the expected number is reached (36 for the intervention group and 36 for the control group). Before the intervention, participants randomly assigned to the intervention group will be divided into three subgroups consisting of 12 individuals. Each subgroup will follow the mindfulness courses (led by the same instructor) for 6 weeks. The courses will be delivered every Monday for subgroup 1, Tuesday for subgroup 2, and Wednesday for subgroup 3. Nurses will attend the intervention during working hours as part of the continuing professional education (CPE) courses provided by the Verona Hospital Trust Administration.

### Assignment of interventions: blinding

Participants will be screened by an independent psychologist after completing the MBI-GS. The randomization will be independently conducted by the trial statistician. The assessments pre-treatment, post-treatment, and at 1-month post-treatment will be completed online. The participants and the treatment psychologist will not be blind.

### Data collection and management

All research data will be retained in the psychiatry section of the University of Verona. Participants scoring above the cut-off score of the screening questionnaire will enter the study and will complete the assessments through a web-based system. Online questionnaire completion time is estimated at approximately 30 min and will be easily carried out using a PC, tablet, or smartphone. The battery of questionnaires will be compiled using the online system Lime Survey (www.limesurvey.com). Data will be stored on the cloud and passwords protected. At the baseline evaluation, each participant will be required to generate a password that should have been used also at the subsequent follow-ups; this was required for the research team to longitudinally link the questionnaires completed by a given nurse at each evaluation point. The study description and the invitation to participate as well as the link to the online questionnaire will be sent via e-mail to all nurses who meet the criteria by the trust administration. A reminder for completing the questionnaire was sent around after one week. The survey will be anonymous, and confidentiality of information will be guaranteed. The data collected will be downloaded and managed by the person responsible for data management and statistical analysis (Chiara Bonetto). Antonio Lasalvia will be responsible for data retention. In accordance with the Declaration of Helsinki, participant confidentiality will be fully preserved throughout the study. It will not be possible to associate the results with the single compiler of the questionnaires or with the single structure to which they refer in any published papers.

### Statistical methods

Statistical analysis will be based on an intention-to-treat (ITT) basis, comparing outcomes from all nurses allocated to the two trial arms. Findings will be reported according to the CONSORT guidelines for parallel-group randomized trials [47].

Personal socio-demographic information and job-related characteristics will be summarized descriptively according to the treatment arms. Categorical data will be presented as frequencies and percentages. Means, standard deviations, medians, interquartile ranges, and minima and maxima will be presented for continuous data. To verify the success of randomization, the baseline characteristics of the two treatment groups will be compared: continuous variables will be compared with a *t*-test (for normally distributed variables) or the Mann-Whitney *U* test (for non-normally distributed variables). Categorical variables will be evaluated with the chi-square or 2-tailed Fisher exact test. Change scores will be calculated. Data will be presented as percentages for categorical data and means and standard deviations for continuous data; 95% CIs will be used to indicate uncertainty around the estimates.

Follow-up data will be analysed using mixed models to establish the correlation between repeated measurements and examine main effects and their interaction, adjusting for the baseline score. The presence of multi-collinearity, interaction, and higher power terms will be assessed to check final model validity. Statistical significance will be defined at two-sided *p* < .05. All analyses will be carried out using Stata 17.0 for Windows.

The effects of baseline covariates expected to have an important influence on the primary outcome will be controlled for by comparing covariate-adjusted analyses with unadjusted analyses. Certain covariates that will be considered at baseline will refer to demographic variables (e.g. gender, age, and education level), the organizational context, and participation in non-mindfulness support courses.

The ITT principle will allow for potential biases arising from loss to follow-up, under the assumption that missing outcomes are missing at random (MAR) using Little and Rubin’s terminology (2002) [48]. Mixed models will allow for the inclusion of data from nurses with incomplete observations at follow-up. We will allow for the presence of missing outcome data on the assumption that the data are missing completely at random, conditional on the covariates included in the models (i.e. missing at random), again using Little and Rubins’ terminology [48]. Multiple imputation methods will be applied in the event of missing data.

### Oversight and monitoring

As has been noted, participants who experience (serious) adverse events and harm resulting from the intervention or those allocated in the waiting list who experience a worsening of their mental health status will be referred to the Psychiatric Consultation Service at the Psychosomatics Unit and the project management group; they, however, will be excluded from the study. The ethics committees will be also informed.

### Dissemination of findings

The results will be presented in international peer-reviewed journals if accepted. The principal investigators, co-investigators, and other professionals involved in the proposed intervention will be the authors of any publications. The criteria established by the International Committee of Medical Journal Editors will be adopted. Entitlement to authorship will be based on the participants’ substantive contribution to the study design, data analysis, and interpretation, the writing of the article, its critical revision, and final approval before submission.

## Discussion

The proposed trial is the first to evaluate the effectiveness of a mindfulness and compassion-focused intervention for the reduction of levels of burnout and distress in nurses working during the COVID-19 pandemic. Mental health in HCWs is a sensitive subject because the pandemic has had serious consequences for them. These include higher levels of burnout, depression, anxiety, insomnia, and post-traumatic symptoms than those of the pre-pandemic period. Several studies indicate that symptomatology did not spontaneously resolve 1 year after the onset of the pandemic [4]. Indeed, mental health outcomes amongst HCWs deteriorated [4]. The most stressful factors include having experienced sudden and dramatic challenges in terms of increased workload, reassignment/redeployment to other roles or duties, infection threat, COVID-19-related traumatic events, and frustration with the death of patients for whom they provided care [2, 4]. An effective and replicable psychosocial intervention must be provided to HCWs who are most in need of help.

It should however be acknowledged that the intervention tested in this research will target only intrapsychic psychological factors of burnout. It is well known that burnout *arises* as a result of multiple factors, where contextual factors play a relevant role. In this regard, to mitigate the effect of burnout it is important that any psychological intervention provided at individual level should be also supplemented by other interventions at the system level aimed at improving working conditions, providing safety and protection in the workplace, and implementing safe staffing ratios and workloads.

The trial presents potential risks. In particular, the sample is characterized by high levels of stress and is therefore at greater risk of psychiatric comorbidities. This may lead to difficulties in recruitment, data collection, and treatment adherence. However, the existing literature on harm in evidence-based mindfulness interventions is sparse, and there is no evidence that suggests any side effects [49].

## Data Availability

The data file will not be publicly available. An anonymous copy of the data file of this study will be available from the corresponding author upon reasonable request.

## Appendix

Table 3

**Table 3:** World Health Organization trial registration data set

Data category	Information
Primary registry and trial identifying number	ClinicalTrials.gov; Identifier: NCT05308537
Date of registration in primary registry	1 April 2022
Secondary identifying numbers	----
Source(s) of monetary or material support	No external funding will be provided. The study will be supported by personal funding of each investigator from the University of Verona
Primary sponsor	No sponsor
Secondary sponsor(s)	No sponsor
Contact for public queries	Prof. Antonio Lasalvia, PhD, MD telephone: + 39 045 8283911 e-mail: antonio.lasalvia@univr.it
Contact for scientific queries	Prof. Antonio Lasalvia, PhD, MD Section of Psychiatry, Department of Neuroscience, Biomedicine and Movement Sciences, University of Verona Policlinico “G.B. Rossi” P.le Scuro, 10 37134 – Verona, Italy
Public title	Evaluating a Mindful Compassion Care Program for reducing burnout and psychological distress amongst frontline hospital nurses during the COVID-19 pandemic
Scientific title	The effectiveness of the Mindful Compassion Care Program (MCCP) in reducing burnout and psychological distress amongst frontline hospital nurses during the COVID-19 pandemic: a study protocol for a randomized controlled trial
Countries of recruitment	Italy
Health condition(s) or problem(s) studied	Psychological intervention, burnout, psychological distress, frontline hospital nurses, COVID-19 pandemic
Intervention(s)	Experimental intervention: Mindful Compassion Care Program Control intervention: Waiting List
Key inclusion and exclusion criteria	Ages eligible for study: ≥ 18 years Sexes eligible for study: both Accepts healthy volunteers: no Inclusion criteria: (1) nurses employed at Verona Academic Hospital Trust over the previous 2 years; (2) working within intensive care units, infectious disease unit, and pulmonary medicine and internal medicine units that have been converted to COVID-19 sub-intensive units; and (3) scoring above the cut-off score for the Emotional Exhaustion sub-scale (EX) of the Maslach Burnout Inventory-General Survey (MB-GS). Exclusion criteria: (1) participation in a mindfulness-based interventions over the previous 6 months; (2) score < 2.20 in the EX subscale of the MBI-GS; and (3) currently receiving psychosocial or psychiatric treatment
Study type	Interventional Allocation: randomized Intervention model: parallel assignment Masking: no blinding Primary purpose: treatment
Date of first enrolment	September 2022
Target sample size	72
Recruitment status	Advertising
Primary outcome(s)	Change in the sub-subscale of emotional exhaustion (EX) of the Maslach Burnout Inventory-General Survey (MBI-GS) between pre-treatment and post-treatment
Key secondary outcomes	Change between pre-treatment and post-treatment on the following dimensions: - Professional efficacy (MBI-GS) - Cynicism (MBI-GS) - Depressive symptoms (PHQ-9) - Anxiety symptoms (GAD-7) - Post-traumatic symptoms (IES-R) - Insomnia (ISI) - Perception of stress (PSS) - Mindfulness skills (FFMQ) - Evaluation of self-criticism and self-reassurance (FSCRS)

### Trial status

The trial will start on September 15, 2022, and continue until December 15, 2022.

## Abbreviations

AOUI: Azienda Ospedaliera Universitaria Integrata
CFT: Compassion-focused therapy
COVID-19: Coronavirus disease 2019
HCWs: Healthcare workers
MBCT: Mindfulness-based cognitive therapy
MCCP: Mindful Compassion Care Program (MCCP)
MBSR: Mindfulness-based stress reduction
MBI-GS: Maslach Burnout Inventory – General Survey
